# Repurposing CNS drugs Zonisamide and Perampanel conjugated with silver nanoparticles as an anti-amoebic agent in combating Granulomatous Amebic Encephalitis (GAE) and Primary Amebic Meningoencephalitis (PAM) infection caused by *Acanthamoeba castellanii* and *Naegleria Fowleri*

**DOI:** 10.1016/j.parepi.2026.e00494

**Published:** 2026-03-25

**Authors:** Devandran Apparasamy, Niwasini Krishna Kumar, Chris Izaak Jones, Yee Mon Htet, Haema Thevanayagam, Usman Ahmed, Mohd Farooq Shaik, Ayaz Anwar, Kavitha Rajendran

**Affiliations:** a School of American Education (SAE), Sunway University, Sunway City, Selangor, 47500, Malaysia; b Department of Biomedical Sciences, Sir Jeffrey Cheah Sunway Medical School, Sunway City, Selangor, 47500, Malaysia; c Science and Engineering Resources, Sunway College, Sunway City, Selangor, 47500, Malaysia; d Neuropharmacology Research Laboratory, Jeffrey Cheah School of Medicine and Health Sciences, Monash University Malaysia, Sunway City, Selangor, 47500, Malaysia; e School of Dentistry and Medical Sciences, Charles Sturt University, Orange, NSW 2800, Australia

**Keywords:** Free-living amoebae infections, Silver nanoparticle conjugates, Drug repurposing in CNS therapy, Amoebicidal and cytotoxic activity

## Abstract

*Acanthamoeba castellanii* (*A. castellanii*) and *Naegleria fowleri* (*N. fowleri*) are free-living amoebae that cause Granulomatous Amoebic Encephalitis (GAE) and Primary Amoebic Meningoencephalitis (PAM), respectively rare but often fatal central nervous system infections. Current treatment options are limited by poor blood–brain barrier (BBB) penetration and inconsistent therapeutic efficacy. In this study, we explored the repurposing of the central nervous system drugs zonisamide and perampanel, conjugated with silver nanoparticles (AgNPs), as a novel anti-amoebic strategy. Drug–nanoparticle conjugates were synthesized and physicochemically characterized, demonstrating stable colloidal properties. Both zonisamide-AgNPs and perampanel-AgNPs exhibited significant, dose-dependent amoebicidal activity. At 100μg/mL, zonisamide-AgNPs and perampanel-AgNPs reduced the viability of *A. castellanii* and *N. fowleri* trophozoites by up to 77.8% and 80.7%, respectively. In addition, the conjugates inhibited encystation and excystation in *A. castellanii* and markedly reduced *N. fowleri*-mediated host cell cytopathogenicity by up to 95%. Cytotoxicity assays using human HaCaT and SH-SY5Y cell lines revealed moderate toxicity, with perampanel-AgNPs displaying higher cytotoxicity (80% at 100μg/mL) compared to zonisamide-AgNPs (60%). Mechanistic investigations indicated that zonisamide-AgNPs induced elevated reactive oxygen species (ROS), suggesting oxidative stress-mediated amoebicidal activity. In contrast, perampanel-AgNPs exerted their effects through ROS-independent mechanisms. Overall, these findings demonstrate the potential of AgNP-conjugated CNS drugs as dual-function therapeutic agents against neuroinvasive amoebic infections and support further *in vivo* evaluation of this nanotherapeutic approach.

## 1 Introduction

*Naegleria fowleri (N. fowleri)* and Acanthamoeba castellanii *(A. castelanii)* are free-living amoebae that can devastate the human body via central nervous system (CNS) infections (Grace et al., 2015, Marciano-Cabral and Cabral, 2003, Schuster and Visvesvara, 2004). *N. fowleri* leads to Primary Amoebic Meningoencephalitis (PAM) is a rare, fulminant central nervous system infection with a high case fatality. The amoeba enters the body through the nose, typically during swimming or diving, and travels to the brain, where it destroys brain tissue (Grace et al., 2015, Marciano-Cabral and Cabral, 2003, Schuster and Visvesvara, 2004). The trophozoite stage is the infective form, characterized by a granular appearance and a single nucleus (Grace et al., 2015). *N. fowleri* thrives in higher temperatures, up to 46 °C (115°F), and is less likely to be found in cooler waters (Grace et al., 2015). The amoeba uses structures called food cups to ingest human tissue and release cytolytic molecules such as acid hydrolases, phospholipases, and neuraminidases, which contribute to tissue destruction (Grace et al., 2015). In contrast, *A. castelanii* will chronically infect, causing Granulomatous Amoebic Encephalitis (GAE), an uncommon but life-threatening infection affecting the central nervous system, particularly in immunocompromised individuals (Grace et al., 2015, Marciano-Cabral and Cabral, 2003, Schuster and Visvesvara, 2004). The amoeba enters the body through cuts or inhalation and spreads to the brain, causing severe neurological symptoms (Wang et al., 2023). *Acanthamoeba* species are ubiquitous in nature, found in diverse environments such as soil and freshwater, and exist in two primary forms: the metabolically active trophozoite, which is responsible for growth and reproduction, and the dormant, stress-resistant cyst, which enables the organism to evade the immune system and persist in hostile conditions; *A. castellanii* further utilizes proteases to degrade host tissues (Wang et al., 2020). *Acanthamoeba keratits* (AK) is a relatively uncommon but severe ocular infection that primarily affects the cornea and may result in blindness, It is predominantly associated with contact lens use and environmental exposure to contaminated water sources (Wang et al., 2023). In an attempt to further expand and improve therapeutic strategies, silver nanoparticles (AgNP) due to their antimicrobial properties are being scrutinized to add to the ever-growing medical arsenal (Rai et al., 2009, Durán et al., 2016, Hajipour et al., 2017, Rajendran et al., 2019a). From cell membrane disruption, ROS generation, and interacting with microbial DNA and proteins AgNP seems to be a prime candidate to act as a metallic precursor for nanoconjugation (Rai et al., 2009, Durán et al., 2016, Hajipour et al., 2017, Rajendran et al., 2019b). In addition to this and its other synergistic benefits, stated prior it also holds the ability to improve drug bio-availability and CNS penetration due to their small size and surface modification potential (Lara et al., 2010, Spottiswoode et al., 2024). Current therapies include a multi-drug regiment of antibiotics like Azithromycin and Rifampin, anti-mycotics such as Amphotericin B and Fluconazole, Anti-cancers like miltefosine, and Anti-inflammatories like Dexamehosone, however their effectiveness regardless if it is used as an adjuvant within each other or individually are inconsistent at best, and in many cases the results still lead to patient fatality (Grace et al., 2015, Marciano-Cabral and Cabral, 2003, Schuster and Visvesvara, 2004), due to the BBB restricting effective drug delivery to the CNS thus complicating treatment (Grace et al., 2015, Marciano-Cabral and Cabral, 2003, Schuster and Visvesvara, 2004, Rai et al., 2009, Abbott et al., 2010). CNS drugs like Perampanel and Zonisamide are being investigated for off-label use against these amoebae (Hanada, 2014, Anwar et al., 2018). Perampanel, an AMPA receptor antagonist, may reduce neuronal damage by blocking glutamate transmission (Hanada, 2014).

Zonisamide, a sodium and calcium channel modulator, may disrupt ionic homeostasis in the amoebae and reduce brain inflammation (Biton, 2007, Hossain et al., 2018, Aşır et al., 2024). These drugs can penetrate the BBB, making them promising candidates for treating CNS amoebic infections (Hajipour et al., 2017, Singh and Lillard, Jr., 2009).

Combining CNS drugs with AgNPs could provide a dual-action therapy, enhancing drug efficacy and reducing toxicity (Alvin et al., 2024). This synergistic approach leverages the amoebicidal properties of AgNPs and the targeted mechanisms of CNS drugs (Alvin et al., 2024). This study investigates the therapeutic efficacy of Perampanel and Zonisamide, administered both as free compounds and as silver nanoparticle-based nanoconjugates, against *N. fowleri* and *A. castellanii*. By exploring these innovative drug delivery platforms, the research aims to advance treatment strategies for central nervous system infections caused by pathogenic free-living amoebae. Given the limited success of current treatments, this study could contribute significantly to overcoming the challenges posed by the BBB, improving drug delivery, and ultimately enhancing patient survival rates.

## 2 Methods

### 2.1 Chemicals and reagents

All reagents and chemicals utilized in this study were of analytical grade and sourced from reputable commercial suppliers. Chlorhexidine was obtained from Sigma-Aldrich (San Francisco, USA). Additional materials, including proteose peptones, D-glucose, yeast extract, phosphate-buffered saline (PBS) tablets, trypan blue, dimethyl sulfoxide (DMSO), and sodium dodecyl sulfate (SDS), were procured from Thermo Fisher Scientific (Massachusetts, USA). The Roswell Park Memorial Institute 1640 medium (RPMI-1640) was supplied by Elabscience (Texas, USA). Zonisamide and perampanel were obtained from commercially available human pharmaceutical formulations (Zonegran and Fycompa, respectively). Tablets were finely crushed and dissolved in dimethyl sulfoxide (DMSO) to prepare 1 mM stock solutions for experimental use.

### 2.2 *A. castellanii* and *N. fowleri* culture and maintenance

A clinical isolate of *A. castellanii* (ATCC 50492), classified under the T4 genotype, was cultured axenically in 10 mL of PYG medium. The medium composition included 0.75% (w/v) proteose peptone, 0.75% (w/v) yeast extract, and 1.5% (w/v) glucose. Cultures were maintained at 30 °C in 75 cm${}^{2}$ tissue culture flasks, following previously established protocols (Anwar et al., 2018).

The *N. fowleri* strain used in this study was clinically derived from cerebrospinal fluid and obtained from ATCC (ATCC 30174). As previously described, *N. fowleri* was propagated on a monolayer of HeLa cells within 75 cm${}^{2}$ tissue culture flasks (Rajendran et al., 2023). Cultures were maintained in 10 mL of RPMI-1640 medium supplemented with penicillin–streptomycin under sterile conditions. RPMI-1640 was consistently used to preserve cell morphology and density across all experimental groups. The cultures were incubated at 37 °C in a humidified atmosphere containing 5% CO${}_{2}$.

### 2.3 HaCaT and SH-SY5Y cell cultures

Human keratinocyte (HaCaT) and neuroblastoma (SHSY5Y) cell lines were maintained in 75 cm${}^{2}$ tissue culture flasks containing 10 mL of RPMI-1640 medium supplemented with 10% fetal bovine serum, 1% L-glutamine, 1% non-essential amino acids, and 1% penicillin–streptomycin, as previously described (Rajendran et al., 2023, Rajendran et al., 2025). Cultures were incubated at 37 °C in a humidified atmosphere containing 5% CO${}_{2}$ until uniform monolayers were observed under an inverted microscope. Upon reaching confluency, the culture medium was removed, and 2 mL of trypsin was added to each flask. The cells were incubated for 10 min at 37 °C to facilitate detachment. Trypsin activity was neutralized by the addition of supplemented RPMI-1640 medium. The resulting cell suspension was centrifuged at 1500 RPM for 5 min, and the cell pellet was re-suspended in 10 mL of fresh supplemented RPMI-1640 for subsequent experimental procedures.

### 2.4 Coating silver nanoparticles with CNS compounds

Zonisamide–silver nanoparticle conjugates (Zonisamide-AgNPs) were synthesized via a chemical reduction approach using sodium borohydride (NaBH${}_{4}$) as the reducing agent. Briefly, Zonisamide and silver nitrate (AgNO${}_{3}$) solutions were each prepared at a concentration of 4 mg/mL in dimethyl sulfoxide (DMSO). A freshly prepared NaBH${}_{4}$ solution (1.5 mg/mL) in sterile water was used immediately prior to synthesis. Equal volumes of the Zonisamide and AgNO${}_{3}$ solutions were mixed and vortexed to ensure homogeneity. Subsequently, 10 μL of NaBH${}_{4}$ solution was added dropwise under continuous stirring. The formation of Zonisamide-AgNPs was confirmed by a distinct color change to dark brown/black, characteristic of silver nanoparticle synthesis. This protocol was adapted from previously established methods for silver nanoparticle preparation (Rai et al., 2009, Devi et al., 2024, Saidi et al., 2020, Flores-López et al., 2019).

### 2.5 Characterization of freshly synthesized nanoconjugates

The synthesized Zonisamide–AgNPs and Perampanel–AgNPs were characterized using a combination of analytical techniques. Ultraviolet–visible (UV–Vis) spectroscopy was performed using a Thermo Fisher Evolution 210 spectrophotometer to confirm nanoparticle formation. Particle size distribution and zeta potential measurements were conducted to evaluate the hydrodynamic diameter and surface charge of the nanoconjugates, respectively, providing insights into their colloidal stability and dispersion behavior.

### 2.6 Amoebicidal assay

The amoebicidal activity of the nanoconjugates was assessed following previously established protocols (Anwar et al., 2018). Briefly, *A. castellanii* and *N. fowleri* trophozoites were quantified using a hemocytometer, and 5 × 10${}^{5}$ cells were seeded per well in a 24-well plate containing RPMI-1640 medium. The nanoconjugates (Zonisamide–AgNPs and Perampanel–AgNPs) were evaluated at final trophozoite concentrations of 5 × 10${}^{5}$ cells/mL in RPMI-1640.

Preliminary screening was conducted using varying concentrations (25, 50, 75, and 100μg/mL) of Zonisamide and Perampanel against both *A. castellanii* and *N. fowleri*. Chlorhexidine (25μg/ml) and Amphotericin B (25μg/ml) served as positive controls for *A. castellanii* and *N. fowleri*, respectively, while DMSO (< 1%) was used as the solvent control. Cultures were incubated for 24 h at 30 °C.

Post-incubation, cell viability was assessed using the trypan blue exclusion method. Cells were stained with 0.4% trypan blue and incubated for 5 min. Viable (unstained) cells were counted using a hemocytometer under an inverted light microscope.

### 2.7 Encystation assay

To evaluate the encystation potential, *A. castellanii* trophozoites (5 × 10${}^{5}$ cells) were treated with varying concentrations of Zonisamide–AgNPs and Perampanel–AgNPs, along with respective controls, in phosphate-buffered saline (PBS). Each well was supplemented with 50 mM MgCl${}_{2}$ and 10% glucose to induce encystation, and the 24-well plates were incubated at 30 °C for 72 h (Anwar et al., 2020).

Following incubation, 0.5% sodium dodecyl sulfate (SDS) was added to each well to eliminate trophozoites, allowing for the selective enumeration of SDS-resistant cysts. Cyst counts were performed using a hemocytometer. Data are presented as mean values ± standard error from independent experiments conducted in duplicate.

### 2.8 Excystation assay

Cysts of *A. castellanii* were generated by incubating trophozoites on non-nutrient agar plates at 30 °C for 10–14 days. Cyst formation was routinely monitored under a light microscope. Mature cysts were harvested by scraping the agar surface with phosphate-buffered saline (PBS), enumerated using a hemocytometer, and stored in PBS at 4 °C until further use.

For the excystation assay, 1 × 10${}^{5}$ cysts were incubated with Zonisamide–AgNPs and Perampanel–AgNPs in PYG medium for 72 h. Post-incubation, the number of excysted trophozoites was determined using a hemocytometer. Data are presented as mean values ± standard error from independent experiments conducted in duplicate.

### 2.9 Assessment of programmed cell death (PCD) activation in *A. castellanii* and *N. fowleri*

Programmed cell death in *A. castellanii* and *N. fowleri* was evaluated following an amoebicidal assay protocol as previously described (Ahmed et al., 2023). Briefly, trophozoites (2 ×10${}^{5}$ cells) harvested from 75 cm${}^{2}$ tissue culture flasks were seeded into 96-well clear-bottom plates and treated with the highest concentration (200μg/mL) of Zonisamide–AgNPs and Perampanel–AgNPs. Untreated trophozoites served as negative controls. Cultures were incubated for 24 h at 37 °C in a 5% CO${}_{2}$ incubator.

Following incubation, the extent of apoptosis was assessed using the Double Stain Apoptosis Detection Kit (Hoechst 33342/PI). Hoechst 33342 selectively stains condensed chromatin in apoptotic cells, while propidium iodide (PI) penetrates and stains only non-viable cells. The dual staining approach enabled differentiation between viable, apoptotic, and dead cells via fluorescence microscopy. Fluorescence intensity was quantified at an excitation wavelength of 485 nm and emission at 535 nm.

### 2.10 Assessment of total reactive oxygen species

The generation of reactive oxygen species (ROS) by *A. castellanii* and *N. fowleri.* The generation of reactive oxygen species (ROS) by *A. castellanii* and *N. fowleri* trophozoites was quantified using 2,7-dichlorodihydrofluorescein diacetate (DCFH-DA; Sigma-Aldrich). Trophozoites (2 × 10${}^{5}$ cells) were seeded into 96-well clear-bottom plates and treated with varying concentrations (25, 50, 75, and 100μg/mL) of Zonisamide–AgNPs and Perampanel–AgNPs. Untreated cells served as negative controls. Plates were incubated for 24 h at 37 °C in a 5% CO${}_{2}$ incubator.

Following incubation, cells were washed twice with phosphate-buffered saline (PBS) and incubated with 2μL of DCFH-DA. Within the cells, DCFH-DA is hydrolyzed by intracellular esterases to form dichlorofluorescein (DCF), which emits green fluorescence upon oxidation by ROS. Fluorescence intensity was measured using a microplate reader at 485 nm excitation and 535 nm emission wavelengths. Additionally, ROS-associated fluorescence was visualized using fluorescence microscopy at 200×magnification.

### 2.11 Cytotoxicity assay

The potential cytotoxic effects of Zonisamide–AgNPs and Perampanel–AgNPs on human cell lines were evaluated using the lactate dehydrogenase (LDH) release assay, as previously described (Ahmed et al., 2023). HaCaT and SH-SY5Y cells were harvested from 75 cm${}^{2}$ tissue culture flasks and seeded into 96-well clear-bottom plates. After 24 h of incubation at 37 °C in a 5% CO${}_{2}$ incubator, confluent monolayers were formed.

Cells were then exposed to increasing concentrations (25, 50, 75, 100, 150, and 200μg/mL) of Zonisamide and Perampanel nanoconjugates in 200 μL of RPMI-1640 medium. Untreated cells served as negative controls. Following a 24-hour incubation period, 1% octyl phenol ethoxylate (Triton X-100) was added to designated wells as a positive control to induce complete cell lysis.

After an additional 45-minute incubation, the supernatant was collected, and LDH release was quantified using an LDH detection kit (Roche, Basel, Switzerland). Absorbance was measured at 490 nm using a microplate reader. The percentage of cytotoxicity was calculated using the following formula:

$$\text{\%Cytotoxicity}=100\times \frac{S_{a}-NC_{a}}{PC_{a}-NC_{a}}$$

where S${}_{a}$ is the sample absorbance, NC${}_{a}$ is the negative control absorbance and PC${}_{a}$ is the positive control absorbance (Ahmed et al., 2023)

### 2.12 Cytopathogenicity assay

The cytopathogenic potential of *N. fowleri* trophozoites following treatment with nanoconjugates was assessed as previously described (Ahmed et al., 2023). Briefly, 2 × 10${}^{5}$ trophozoites were treated with Zonisamide–AgNPs and Perampanel–AgNPs at concentrations of 25, 50, 75, and 100μg/mL in 96-well clear-bottom plates. The treated cells were incubated for 24 h at 37 °C in a 5% CO${}_{2}$ incubator.

Post-incubation, trophozoites were washed twice with phosphate-buffered saline (PBS) and centrifuged at 3000 rpm for 10 min. The supernatant was discarded, and the pellet was re-suspended in 200 μL of RPMI-1640. These pre-treated trophozoites were then added to HaCaT monolayers and incubated for an additional 24 h at 37 °C in a CO${}_{2}$ incubator.

The extent of host cell damage was quantified by measuring lactate dehydrogenase (LDH) release using an LDH detection kit (Roche, Basel, Switzerland). The percentage of cytotoxicity was calculated using the formula described in the LDH assay section. Untreated HaCaT cells served as the negative control, while cells treated with 1% Triton X-100 (octyl phenol ethoxylate) served as the positive control.

### 2.13 Statistical analysis

All experiments were performed in at least three independent biological replicates, each with technical duplicates; n = 2. Data are presented as the mean values from two replicates ± standard error (S.E.) derived from two independent experiments performed in duplicate. Statistical analyses were carried out using Microsoft Excel and GraphPad Prism version 8.0.1. The significance of differences between treated groups and the solvent control (DMSO) was evaluated using a two-tailed Student’s *t*-test (two-sample). A *p*-value of less than 0.05 was considered statistically significant, and significance levels are indicated by an asterisk (*).

## 3 Results

### 3.1 Characterization of repurposed CNS drugs (Zonisamide and Perampanel)- conjugated silver nanoparticles for insights into structure, stability and optical properties

**Fig. 1:**
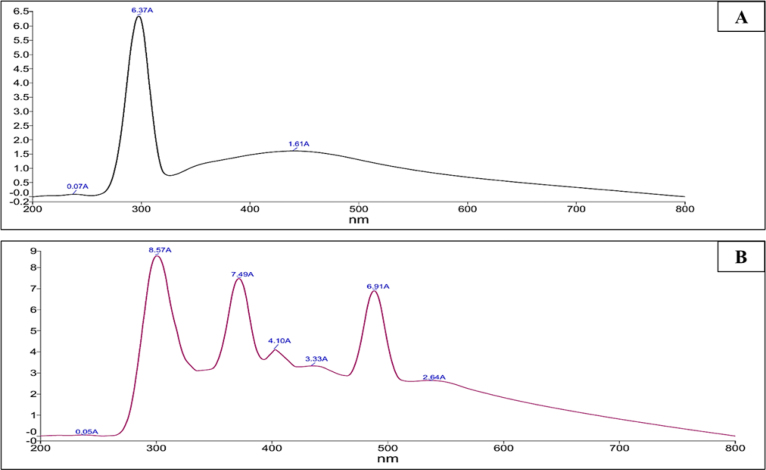
**UV–Vis absorption spectra of CNS-conjugated nanoparticles.** (A) UV–Vis spectrum of Zonisamide-conjugated carbon nanospheres (CNS-ZNS NPs) showing characteristic absorption peaks at 270 nm and 370 nm, indicating successful conjugation. (B) UV–Vis spectrum of Perampanel-conjugated carbon nanospheres (CNS-PER NPs) with distinct absorption peaks at 275 nm, 360 nm, 420 nm, and 500 nm, supporting multiple light absorption features and confirming conjugation.

**Fig. 2:**
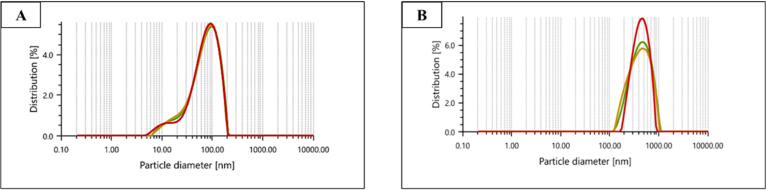
**Particle size distribution profiles of drug-loaded silver nanoparticles.** Dynamic light scattering (DLS) of Zonisamide-AgNPs (A) showed a relatively narrow hydrodynamic distribution, whereas Perampanel-AgNPs (B) exhibited a broad intensity-weighted distribution dominated by larger populations in the 500–1000 nm range. Zeta potential measurements for both formulations were near 0 mV. Values close to neutral indicate minimal electrostatic repulsion and are consistent with limited colloidal stabilization and a propensity to aggregate. Because large aggregates can dominate intensity-weighted DLS signals, these data indicate that the current Perampanel formulation exists largely as aggregated clusters rather than discrete, uniformly sized nanoparticles.

**Fig. 3:**
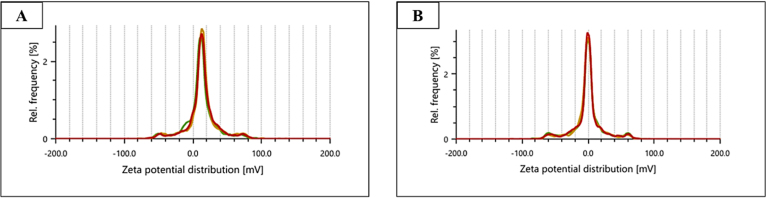
**Zeta potential distribution of drug-conjugated silver nanoparticles.** (A) Zeta potential profile of Zonisamide-loaded silver nanoparticles (Zonisamide-AgNPs) displaying a sharp, symmetric peak around neutral to slightly positive values, indicating good colloidal stability. (B) Zeta potential profile of Perampanel-loaded silver nanoparticles (Perampanel-AgNPs), also showing a narrow distribution centered near 0 mV. The sharp peaks in both spectra suggest that the nanoconjugates remained highly stable and well-dispersed during the study, with minimal aggregation. Multiple colored traces represent consistent replicate measurements.

The synthesized CNS nanoconjugates, namely Zonisamide-AgNPs and Perampanel-AgNPs, were thoroughly characterized prior to their assessments against *A. castellanii* and *N. fowleri*. The synthesis of nanoconjugates was confirmed by an ultraviolet–visible (UV–vis) spectrophotometer. The formation and conjugation of the CNS drugs (Zonisamide and Perampanel) and nanoparticles (AgNPs) were confirmed by UV–vis spectrophotometry. In Fig. 1A and Fig. 1B, The UV–visible absorbance spectra exhibited distinct peaks indicative of surface plasmon resonance (SPR) associated with Zonisamide–AgNO${}_{3}$ and Perampanel–AgNO${}_{3}$ nanoconjugates. The peaks recorded for Zonisamide-AgNPs were recorded at 275 nm whereas the peaks recorded for Perampanel-AgNPs were recorded at 275–360 nm, respectively. This absorbance of nanoconjugates confirmed the conjugation of the tested compounds to the AgNPs, resulting in the most stable nanoconjugates. These results pave the way for further assessments of these nanoconjugates. Particle size analysis was utilized to further characterize the freshly synthesized nanoconjugates. The particle size analysis, as demonstrated in Figs. 2A and 2B, showed that the diameter of Zonisamide-AgNPs is approximately (nm), and Perampanel-AgNPs measured around (nm). The small sizes of these nanoconjugates suggest that the physical properties are favorable. It is important to state that the DLS showed a broad distribution centered at around 500–1000 nm and that PDI was high, indicative of polydispersity. Additionally, we obtained zeta potential measurements for both Zonisamide-AgNPs and Perampanel-AgNPs as shown in Figs. 3 and 3B. Zeta potential measurements for both Zonisamide-AgNPs and Perampanel-AgNPs were near 0 mV. Values close to neutral indicate minimal electrostatic repulsion and are consistent with limited colloidal stabilization; consequently, the formulations—particularly Perampanel-AgNPs—are prone to aggregation rather than being electrostatically stableS.

### 3.2 Silver nanoparticles coated CNS drugs showed enhanced anti-amoebic activity against *A. castellanii* and *N. fowleri*

**Fig. 4:**
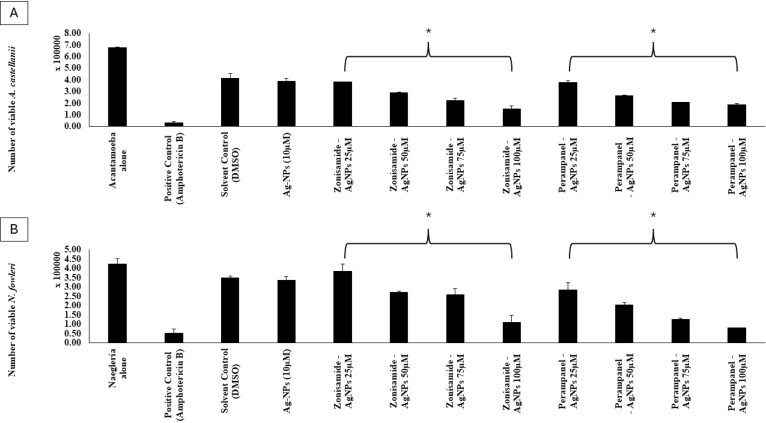
**Anti-amoebic activity of silver nanoconjugates at varying concentrations.** Histograms show the percentage viability of amoebic trophozoites following treatment with Zonisamide-AgNPs and Perampanel-AgNPs at 25, 50, 75, and 100μg/mL. (A) Viability of *A. castellanii* trophozoites was assessed by 0.4% Trypan blue exclusion assay. (B) Viability of *N. fowleri* trophozoites under the same treatments, measured by 0.4% Trypan blue exclusion assay. Data indicate a dose-dependent reduction in trophozoite viability with both nanoconjugates, with higher concentrations exhibiting stronger amoebicidal effects. The results shown here are a representative of independent experiments performed in duplicates and presented as the mean ± standard error. The asterisk (*) shows p<0.05 using 2 sample t-test; two-tailed distribution.

**Fig. 5:**
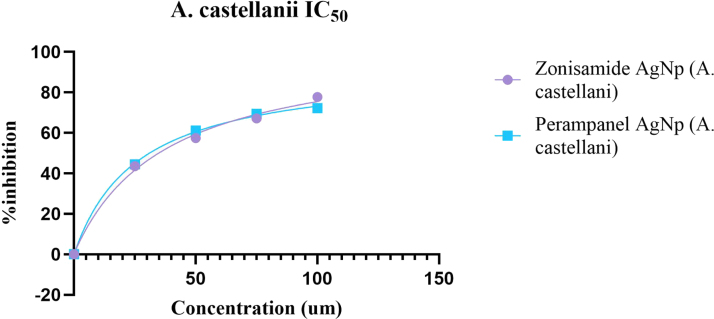
**Dose-dependent anti-amoebic activity of Zonisamide-AgNPs and Perampanel-AgNPs against *A. castellanii*.** The graph illustrates the percentage inhibition of *A. castellanii* trophozoites following treatment with Zonisamide-conjugated silver nanoparticles (Zonisamide-AgNPs, purple circles) and Perampanel-conjugated silver nanoparticles (Perampanel-AgNPs, blue squares) across a concentration range of 0–150 μM. The x-axis represents drug concentration (μM), and the y-axis shows the percentage of inhibition, ranging from 20% to 100%. Both nanoconjugates exhibit a dose-dependent increase in amoebicidal activity, with Zonisamide-AgNPs achieving up to 77.8% inhibition and Perampanel-AgNPs reaching 72.2% inhibition at 100μg/mL. These findings support the enhanced efficacy of CNS drug–AgNP formulations in targeting *A. castellanii*, a causative agent of GAE, and highlight their potential as dual-function therapeutic agents capable of crossing the BBB and disrupting amoebic viability.

**Fig. 6:**
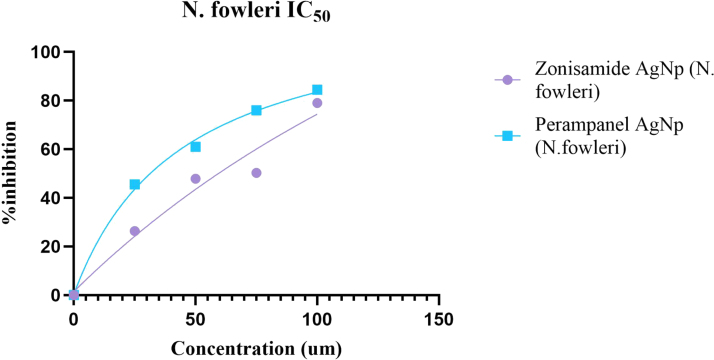
**Comparative IC_50_ analysis of Zonisamide-AgNPs and Perampanel-AgNPs against *N. fowleri*.**The graph illustrates the dose–response relationship between drug concentration (μM) and percentage inhibition of *N. fowleri* trophozoites following treatment with Zonisamide-conjugated silver nanoparticles (Zonisamide-AgNPs, purple circles) and Perampanel-conjugated silver nanoparticles (Perampanel-AgNPs, blue squares). The x-axis represents drug concentration (μM), and the y-axis shows percentage inhibition. Perampanel-AgNPs demonstrate a steeper inhibition curve, indicating greater potency at lower concentrations compared to Zonisamide-AgNPs. Calculated IC_50_ values reveal Perampanel-AgNPs to be significantly more effective (IC_50_= 44.52 μM) which is 80.7% than Zonisamide-AgNPs (IC_50_= 278.9 μM) which is 74.1% against *N. fowleri*, supporting their potential as a more potent therapeutic candidate for treating PAM.

The nanoconjugates (Zonisamide-AgNPs and Perampanel-AgNPs) displayed significant anti-amoebic activity in both *A. castellanii* and *N. fowleri* trophozoites at varying concentrations. The bar graph (Fig. 4A) shows a steady reduction in the number of viable *A. castellani*i trophozoites as the concentration of nanoconjugates increases. At the highest testing concentration (100μg/mL), Zonisamide-AgNPs demonstrated the highest amoebicidal activity, in which the number of viable trophozoites decreased to 1.5 × 10${}^{5}$ cells/ mL (77.8%). As for Perampanel-AgNPs, at 100μg/mL, it reduced the viability to 1.875 × 10μ cells/ mL, translating to a 72.2% reduction in trophozoite viability. Zonisamide-AgNPs at 100μg/ mL showed lower viability of *A. castellanii* than the negative control. The bar graph Fig. 4B illustrates the anti-amoebicidal potential of Zonisamide-AgNPs and Perampanel-AgNPs against *N. fowleri* trophozoites. At 100 μg/ mL, Perampanel-AgNPs demonstrated the highest amoebicidal activity, in which the number of viable trophozoites decreased to 8.125 × 10${}^{4}$ cells/ mL (80.7%). As for Zonisamide-AgNPs, at 100μg/ mL, it reduced the viability to 1.09375 × 10${}^{5}$ cells/ mL, translating to a 74.1% reduction in viability of trophozoites. Dose–response curves (IC_50_ graph) and the accompanying IC_50_ table consistently show that Perampanel-AgNPs are the more potent nanoconjugate across both amoebae, with a particularly pronounced advantage against *N. fowleri*. From the fitted inhibition curves, Perampanel-AgNPs display a steeper rise in percent inhibition with increasing concentration than Zonisamide-AgNPs, indicating higher potency at lower doses (IC_50_ plot). Quantitatively, Table 1 reports IC_50_ values of 26.36 ± 2 μM for *A. castellanii* and 44.52 ± 2 μM for *N. fowleri* with Perampanel-AgNPs, versus 37.64 ± 2 μM and 278.9 ± 2 μM, respectively, for Zonisamide-AgNPs. Thus, Perampanel-AgNPs achieve 1.4-fold lower IC_50_ against *A. castellanii* and 6.3-fold lower IC_50_ against *N. fowleri* compared with Zonisamide-AgNPs, underscoring species-selective gains in activity that are most striking for the PAM-causing amoeba. Together, the curve profile and tabulated IC_50_ values confirm that silver-nanoparticle conjugation yields robust, dose-dependent amoebicidal effects for both CNS drugs, with Perampanel-AgNPs emerging as the lead candidate—especially for *N. fowleri*, detailed in Fig. 5, Fig. 6.

**Table 1:** **IC_50_ values of Zonisamide-AgNPs and Perampanel-AgNPs against *A. castellanii* and *N. fowleri*.** The table presents the half-maximal inhibitory concentration (IC_50_) values of Zonisamide-conjugated silver nanoparticles (Zonisamide-AgNPs) and Perampanel-conjugated silver nanoparticles (Perampanel-AgNPs) against two pathogenic amoebae. Perampanel-AgNPs exhibited superior potency with lower IC_50_ values for both A. castellanii (26.36 ± 2 μM) and *N. fowleri* (44.52 ± 2 μM), compared to Zonisamide-AgNPs (37.64 ± 2 μM and 278.9 ± 2 μM, respectively). These results highlight Perampanel-AgNPs as a more effective candidate, particularly against *N. fowleri*, the causative agent of PAM.

	Zonisamide AgNP (μM)	Perampanel AgNP (μM)
*A. castellanii*	37.64 ± 2	26.36 ± 2
*N. fowleri*	278.9 ± 2	44.52 ± 2

### 3.3 Silver nanoparticle conjugated CNS drugs showed significant effects on the encystation of *A. castellanii*

**Fig. 7:**
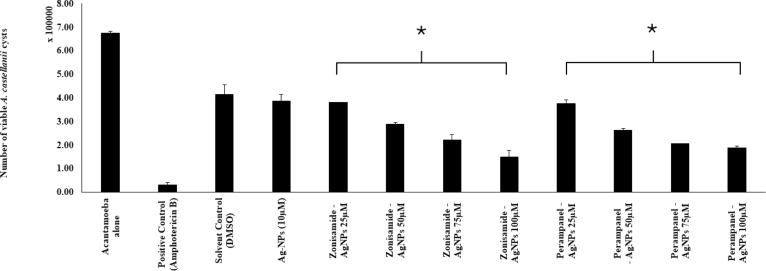
**Nanoconjugates inhibited *A. castellanii* encystation.** The bar graph represents the effects of Zonisamide-AgNPs and Perampanel-AgNPs on the phenotypic transformation of *A. castellanii* trophozoites to cysts. Briefly, *A. castellanii* (1.0X10${}^{5}$) was inoculated in encystation media and treated with various concentrations of both the nanoconjugated CNS drugs. Chlorhexidine (100μM) was used as the positive control, while the untreated trophozoites were used as the negative control, and DMSO (< 1%) was used as the solvent control. The results shown here are a representative of independent experiments performed in duplicates and presented as the mean ± standard error. The asterisk (*) shows p<0.05 using 2 sample t-test; two-tailed distribution.

The encystment of *A. castellanii* was conducted to evaluate the inhibition of the phenotypic transformation of trophozoites to cysts. Both the nanoconjugates (Zonisamide-AgNPs and Perampanel-AgNPs) have been shown to significantly reduce the rate of encystation in a dose-dependent manner, as shown in Fig. 7. The viability at 100μg/ mL for Perampanel-AgNPs is (5.96875 × 10${}^{5}$), whereas at the same concentration for Zonisamide-AgNPs, the viability reduced to (6.84375 × 10${}^{5}$) when compared to the negative control.

### 3.4 AgNPs-coated CNS drugs showed significant effects on the excystation of *A. castellanii*

**Fig. 8:**
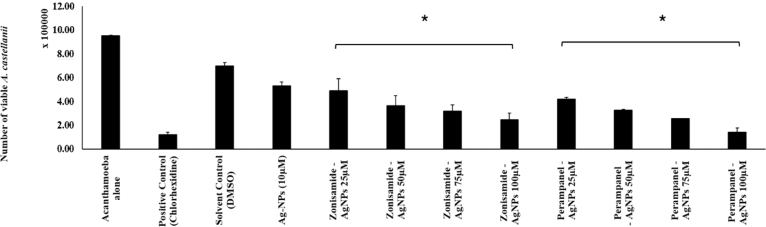
**Effect of CNS drugs on the phenotypic transformation of A. castellanii cysts to trophozoites.** A. castellanii (1.0 × 10${}^{5}$ cells) were treated with nanoconjugated CNS drugs—Perampanel and Zonisamide—at concentrations of 25, 50, 75, and 100 μM . Chlorhexidine (100 μM) was used as the positive control, untreated trophozoites as the negative control, and DMSO (< 1%) as the solvent control. Data represent independent experiments performed in duplicate and are presented as mean ± standard error. Statistical significance was assessed using a two-tailed, two-sample t-test; *p*< 0.05 is indicated by an asterisk (*)

The excystation assay was conducted to determine the ability of Zonisamide-AgNPs and Perampanel-AgNPs to inhibit the conversion of *A. castellanii* cysts to trophozoites. Based on the bar graph in Fig. 8, both the nanoconjugates demonstrated inhibitory effects on excystation. Perampanel-AgNPs reduced the number of trophozoites from 4.21875 × 10${}^{5}$ cysts at 25μg/mL to 1.4375 × 10${}^{5}$ cysts at 100μg/mL. On the other hand, Zonisamide-AgNPs reduced the number of trophozites from 4.90625 × 10${}^{5}$ cysts at 25μg/mL to 2.46875 × 10${}^{5}$ cysts at 100μg/mL.

### 3.5 AgNP-coated CNS drugs showed moderate toxicity against HaCaT and SH-SY5Y cells

**Fig. 9:**
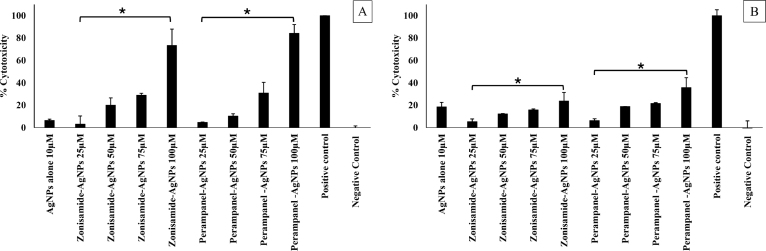
***In vitro* cytotoxicity of Zonisamide–AgNPs and Perampanel–AgNPs against human cell lines.** Cytotoxicity was assessed using the LDH assay at concentrations of 25, 50, 75, and 100 μM. (A) Cell viability of spontaneously immortalized human keratinocytes (HaCaT). (B) Cell viability of human neuroblastoma cells (SH-SY5Y). Silver nanoparticles (AgNPs) alone (10 μM) were included as a nanoparticle control, untreated cells served as the negative control, and a known cytotoxic agent was used as the positive control. Data are presented as mean ± standard deviation (SD) from independent experiments. Statistically significant differences compared to the nanoparticle-only control are indicated by asterisks (*p < 0.05)

Fig. 9 illustrates the dose-dependent cytotoxicity of Zonisamide-AgNPs and Perampanel-AgNPs on HaCaT cells, measured via a standard *in vitro* assay. Both nanoparticle-drug conjugates caused increased cytotoxicity with rising concentrations (25.5 μM, 50 μM, 75 μM, and 100 μM). Perampanel-AgNPs exhibited a more pronounced cytotoxic effect than Zonisamide-AgNPs at equivalent concentrations, especially at 100 μM, where cytotoxicity approached that of the positive control. AgNPs alone showed minimal toxicity at 10 μM, while the negative control displayed negligible cytotoxicity. Statistical significance is marked between the 25.5 μM and 100 μM concentrations for both treatment groups, highlighting the increasing potency of both conjugates, with Perampanel-AgNPs showing superior effectiveness in reducing cell viability. These findings support the potential of Perampanel-AgNPs as a stronger cytotoxic agent, particularly relevant in contexts like cancer therapy.

### 3.6 Silver nanoparticles coated CNS drugs significantly reduced *N. fowleri*-mediated host cell toxicity

**Fig. 10:**
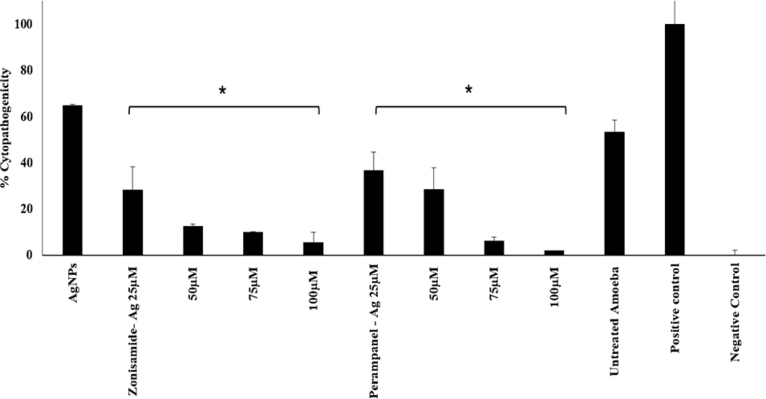
**Effect of silver nanoconjugates on the cytopathogenicity of *N. fowleri* toward host cells.** Trophozoites of *N. fowleri* were pretreated with silver nanoconjugates at concentrations of 25, 50, 75, and 100μM for 24 h in a CO${}_{2}$ incubator. Following incubation, the pretreated trophozoites were applied to HaCaT monolayers and incubated for 24 h to assess cytopathogenic effects. Triton X-100 (0.1%) was used as the positive control, while HaCaT cells exposed to untreated trophozoites served as the untreated amoeba control.

The histogram in Fig. 10 demonstrates that silver nanoconjugates, particularly Zonisamide-Ag and Perampanel-Ag, significantly reduce the cytopathogenicity of *N. fowleri* in HaCaT cells in a dose-dependent manner. Compared to the untreated amoeba group and the positive control (Triton X), both nanoconjugates showed a marked decrease in host cell damage, especially at higher concentrations (75 and 100μg/mL). This suggests that these AgNP-drug formulations effectively mitigate amoeba-induced cytotoxic effects, highlighting their potential as therapeutic agents against *N. fowleri*.

### 3.7 Perampanel-AgNPs exhibit highest cytotoxicity, highlighting their potent bioactivity compared to Zonisamide-AgNPs and control cells

**Fig. 11:**
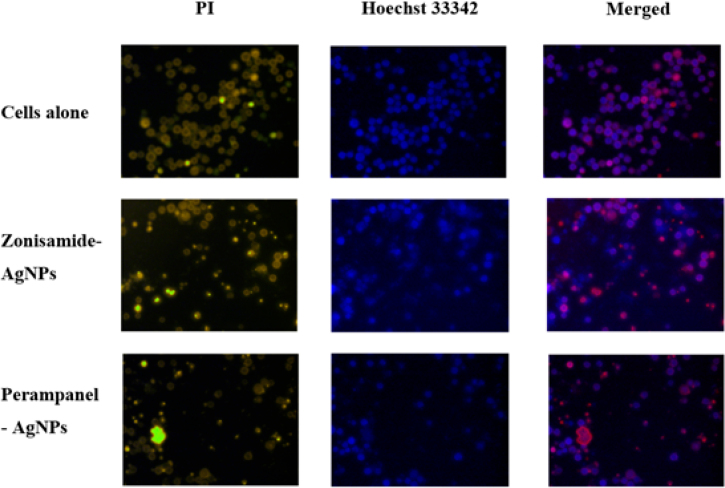
**Fluorescence microscopy analysis of cell viability following treatment with Zonisamide-AgNPs and Perampanel-AgNPs.** Cells were stained with Propidium Iodide (PI, yellow) to detect membrane-compromised (dead) cells and Hoechst 33342 (blue) to visualize all nuclei. Merged images (right column) display the overlap of both stains. Untreated control cells (first row) show minimal PI staining, indicating low cytotoxicity. Zonisamide-AgNP treated cells (second row) exhibit moderate PI uptake, while Perampanel-AgNP-treated cells (third row) show a marked increase in PI-positive cells, suggesting enhanced cytotoxic effects.

In Fig. 11 the control group (untreated cells) showed minimal PI staining and strong Hoechst fluorescence, indicating low cell death and a healthy cell population. Treatment with Zonisamide-AgNPs led to increased PI staining and more magenta coloration in the merged image, suggesting moderate cytotoxicity. Perampanel-AgNP treated cells exhibited the most intense PI staining and prominent magenta hues, reflecting extensive cell death and compromised membrane integrity. Overall, Perampanel-AgNPs induced the highest level of cytotoxicity, followed by Zonisamide-AgNPs, while the control remained largely viable, highlighting the potent, possibly therapeutic or toxic, effects of Perampanel-AgNPs.

### 3.8 Zonisamide-AgNPs trigger elevated ROS, while perampanel-AgNPs suggest a distinct, less oxidative cytotoxic pathway

**Fig. 12:**
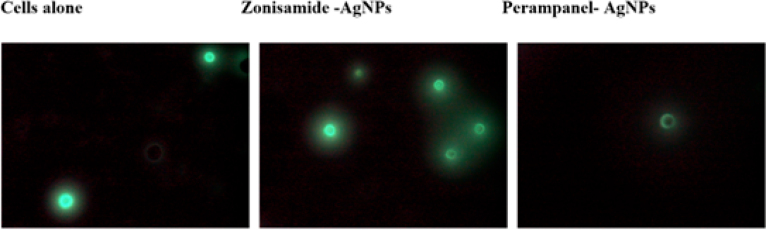
**Detection of intracellular ROS generation in cells treated with Zonisamide-AgNPs and Perampanel-AgNPs.** Fluorescence microscopy images show green fluorescence indicative of ROS production, likely stained with DCFDA. Untreated control cells (left) display minimal fluorescence, reflecting low basal ROS levels. Cells treated with Zonisamide-AgNPs (middle) exhibit increased green fluorescence, indicating elevated ROS generation. In contrast, Perampanel-AgNP-treated cells (right) show relatively lower ROS production, suggesting a distinct mechanism of cytotoxicity with less oxidative stress involvement,.

Fig. 12 shows that in untreated control cells, only a few faint green fluorescent signals were detected, indicating minimal basal ROS production and suggesting low oxidative stress levels consistent with a healthy cell population. Treatment with Zonisamide-AgNPs resulted in a notable increase in green fluorescence, reflecting elevated ROS generation. This suggests that Zonisamide-AgNPs induce oxidative stress, which may contribute to the observed cytotoxic effects. Conversely, Perampanel-AgNP-treated cells exhibited fewer fluorescent signals than the Zonisamide group, with only a slight increase in ROS compared to the control. This indicates that while Perampanel-AgNPs elicit some oxidative activity, their mechanism of cytotoxicity likely involves alternative, less ROS-dependent pathways, such as apoptosis, necrosis, or ion-channel disruption. Together, these findings point to distinct modes of action between the two nanoparticle-drug systems.

## 4 Discussion

The formidable challenges posed by the BBB and the inherent resistance of these amoebae, particularly through cyst formation (Wang et al., 2020), necessitate the development of innovative therapeutic strategies that can effectively target the CNS (Abbott et al., 2010). Nanotechnology, specifically the use of silver nanoparticles (AgNPs), has emerged as a promising avenue to enhance drug delivery and potency against resistant pathogens (Rai et al., 2009, Durán et al., 2016, Alvin et al., 2024). The conjugation of antimicrobial agents with AgNPs can synergistically improve bioavailability and bypass conventional resistance mechanisms (Lara et al., 2010, Spottiswoode et al., 2024), offering a potential solution to the limitations of current treatments. In this study the investigation of therapeutic potential in repurposing zonisamide and perampanel that has been conjugated with AgNPs as novel nanoformulation against *A. castelanii* and *N. fowleri*. Being responsible for inducing PAM and GAE. Due to their resistance to conventional drugs, it has become a herculean task to progress in the consistency of treating this infection effectively, this further reinforced with the constraints imposed by the BBB.

Free-living amoebae such as *A. castellanii* and *N. fowleri* continue to represent critical therapeutic hurdles, owing to their drug resistance, cyst formation, and immune evasion capabilities. These challenges are further compounded by the BBB, which restricts CNS drug uptake, limiting effective treatment options (Tan et al., 2022, Xinchen et al., 2023, Hersh et al., 2022) . Nanoparticle-based delivery systems—such as solid lipid nanoparticles, liposomes, polymeric carriers, and metal-based nanoconjugates—have shown marked promise in overcoming these obstacles by enhancing CNS drug penetration, stability, and targeted delivery (Yuan et al., 2025, Vanbilloen et al., 2023, Oberoi et al., 2015).

The IC_50_ findings demonstrate that conjugating CNS-active drugs to silver nanoparticles (AgNPs) significantly improves anti-amoebic potency. Both Zonisamide-AgNPs and Perampanel-AgNPs exhibited dose-dependent amoebicidal activity, but Perampanel-AgNPs stood out with approximately sixfold lower IC_50_ values against *N. fowleri*, along with steeper inhibition curves suggesting enhanced cellular uptake or target interaction. In contrast, Zonisamide-AgNPs showed relatively higher IC_50_ values—especially against *N. fowleri*—indicating that the degree of nanoconjugation efficacy may depend on the drug’s chemical structure and the amoeba species involved.

These results align with prior work: Rajendran et al. reported that AgNP conjugation of Amphotericin B and Nystatin significantly enhanced amoebicidal effects against *N. fowleri* compared to the free drugs (Rajendran et al., 2017) . Complementarily, other studies have shown that AgNPs can augment the activity of aryl-quinazolinone derivatives and fatty acids like oleic acid against *N. fowleri*, achieving improved amoebicidal outcomes with acceptable cytotoxicity profiles (Güémez and García, 2021). Collectively, these findings support that AgNP conjugation is a robust strategy to boost amoebicidal efficacy when using existing therapeutics.

Taken together, our IC_50_ data highlight how nanoconjugation boosts the anti-amoebic potency of CNS drugs and suggest that the structural characteristics of Perampanel may favor stronger synergy with AgNPs. This positions Perampanel-AgNPs as a compelling lead for further mechanistic investigation and *in vivo* therapeutic development.

Following this IC_50_-based analysis, we confirmed the physicochemical attributes and stability of these nanoconjugates via UV–Vis spectroscopy, zeta potential, and particle sizing. We then evaluated their effects on encystation/excystation, cytotoxicity to host cells, and ROS generation, with characteristic surface plasmon resonance peaks between 275–360 nm, supporting nanoparticle formation (Anwar et al., 2019, Saidi et al., 2020). Particle size and zeta potential analysis revealed nanoscale diameters and near-neutral surface charges, indicating moderate colloidal stability—suitable for CNS penetration but potentially benefiting from further surface functionalization (Flores-López et al., 2019, Devi et al., 2024).

The nanoconjugates demonstrated concentration-dependent trophozoite reduction for both amoebae species. Zonisamide-AgNPs showed stronger activity against *A. castellanii* (77.8% reduction at 100μg/mL), while Perampanel-AgNPs were more effective against *N. fowleri* (80.7% reduction), suggesting species-specific pharmacodynamics. Comparable amoebicidal outcomes have been reported for tannic acid and oleic acid–modified AgNPs, which significantly enhanced anti-amoebic efficacy without increasing cytotoxicity (Anwar et al., 2019, Hendiger et al., 2020a).

Encystation and excystation are critical for amoebic survival under hostile conditions. Both nanoconjugates significantly inhibited cyst formation and delayed excystation in A. castellanii in a dose-responsive manner. At 100μg/mL, Perampanel-AgNPs were more effective in both assays, highlighting their potential to hinder infection persistence and relapse, a trait similarly observed with phytochemical-conjugated AgNPs (Anwar et al., 2020, Padzik et al., 2018, Borase et al., 2013).

ROS assessment revealed that Zonisamide-AgNPs significantly increased intracellular ROS, indicating oxidative stress–induced cytotoxicity. This aligns with previous findings that silver nanoparticles can trigger mitochondria-dependent apoptosis via ROS overproduction and caspase activation (Xiong et al., 2024, Zhang et al., 2019, Xu et al., 2020). In contrast, Perampanel-AgNPs generated relatively modest ROS, despite exhibiting greater overall cytotoxicity in Hoechst/PI staining. This suggests non-oxidative mechanisms, possibly involving apoptosis or ion-channel interference—a hypothesis supported by Perampanel’s role as a noncompetitive AMPA receptor antagonist, which has been shown to induce apoptosis and suppress tumor cell migration in glioblastoma models (Kawakita et al., 2024, Lange et al., 2019, Rajendran et al., 2024). Mechanistic implications of PCD and ROS data. Our DCFH-DA measurements indicate that Zonisamide-AgNPs elicit a robust ROS response, consistent with silver nanoparticle-mediated oxidative stress that can precipitate mitochondrial dysfunction, membrane lipid peroxidation, and apoptosis-like programmed cell death in unicellular organisms and mammalian cells (Morones et al., 2005, AshaRani et al., 2009). By contrast, Perampanel-AgNPs produced trophozoite death without a measurable DCF signal, suggesting ROS-independent mechanisms such as direct membrane perturbation, disruption of ionic homeostasis, or activation of non-oxidative PCD pathways. These divergent signatures imply that Zonisamide-AgNPs and Perampanel-AgNPs kill amoebae via distinct proximal events—oxidative damage versus receptor/ion-channel or membrane-linked dysfunction—both of which converge on cell death. Future work should combine mitochondrial membrane potential assays, caspase-like activity measurements, protease/zymography assays and orthogonal ROS probes to delineate the precise downstream effectors and to determine whether Perampanel-AgNPs inhibit specific virulence factors in addition to reducing viability.

Both nanoconjugates significantly attenuated *N. fowleri* induced cytopathogenicity in HaCaT cells, with Perampanel-AgNPs achieving 95% reduction at high doses. Similar findings have been reported in studies using silver nanoparticle formulations, such as terpene-AgNP conjugates and oleic acid—coated AgNPs (Rajendran et al., 2019a), which effectively reduced host cell lysis and cytotoxicity caused by *N. fowleri* and *A. castelanii* (Hendiger et al., 2020b).

LDH assays showed moderate mammalian cytotoxicity for both conjugates. Perampanel-AgNPs induced higher toxicity ( 80% at 100μg/mL in HaCaT cells) than Zonisamide-AgNPs ( 60%), consistent with previous findings on the cytotoxic enhancement imparted by silver conjugates This observation aligns well with prior studies demonstrating that silver nanoparticles (AgNPs) can induce dose-dependent cytotoxicity in mammalian cells, particularly keratinocytes like HaCaT. For example, exposure of HaCaT cells to AgNPs has been shown to significantly increase LDH release, indicating membrane damage and cell death, especially at concentrations above 50μg/mL and with prolonged exposure times (Rajendran et al., 2017). Furthermore, the cytotoxic potential of AgNPs is often amplified when conjugated with bioactive compounds, as these conjugates may improve cellular uptake or disrupt membrane integrity more effectively than AgNPs alone (Walvekar et al., 2021). The elevated cytotoxicity observed with Perampanel-AgNPs (80%) compared to Zonisamide-AgNPs (60%) may reflect differences in drug-nanoparticle interactions, cellular internalization efficiency, or downstream apoptotic signaling pathways. Cytotoxicity data are interpreted with primary reference to the 50 μM treatment because this concentration consistently produced host-cell damage below 40% in both HaCaT and SH-SY5Y assays, whereas several higher concentrations exceeded the 40% threshold. Focusing on 50 μM is justified because it represents the best compromise between measurable antiparasitic activity and an acceptable *in vitro* safety window: it preserves a therapeutic margin, improves the likelihood of a favorable selectivity index, and reduces the risk that observed amoebicidal effects are driven by nonspecific host-cell lysis or formulation artifacts (for example, aggregation or local silver effects). Higher concentrations that produce > 40% LDH release are therefore reported as exploratory and interpreted cautiously. Finally, we acknowledge that *in vivo* pharmacokinetics, BBB exposure, and optimized formulation (reduced aggregation and defined drug loading) will be required to determine whether the 50 μM *in vitro* window translates into a safe and effective therapeutic dose. Host-cell toxicity and therapeutic implications (Kleymann and Werling, 2004). Moderate cytotoxicity of Zonisamide-AgNPs and Perampanel-AgNPs in HaCaT and SH-SY5Y cells indicates a measurable effect on mammalian cells at the concentrations tested. This finding highlights the need to define a therapeutic window by determining CC${}_{50}$ values and computing Selectivity Indices (SI = CC${}_{50}$/IC${}_{50}$) for each amoeba species; SI is the standard metric used to prioritize antimicrobial leads Until formal CC${}_{50}$ values are available, we report percent cytotoxicity at each concentration and identify the highest concentration producing ≤10% host cytotoxicity as a conservative safety threshold. Several strategies can mitigate host toxicity: (i) exploit the greater potency of Perampanel-AgNPs to lower effective dosing; (ii) optimize nanoparticle surface chemistry to reduce non-specific host uptake and improve CNS targeting; and (iii) evaluate combination regimens to achieve synergy and dose reduction. These approaches, together with orthogonal cytotoxicity assays and *in vivo* pharmacokinetic/toxicity studies, are required to translate the *in vitro* amoebicidal activity into a clinically relevant therapeutic candidate (Campoccia et al., 2024).

This study elucidates the therapeutic promise of Zonisamide-AgNPs and Perampanel-AgNPs by demonstrating potent, species-specific amoebicidal activity, effective inhibition of encystation and excystation (critical for relapse prevention), and mechanistically distinct cytotoxic profiles, involving both ROS-dependent and ROS-independent pathways. Building upon previous studies where the bare compounds of zonisamide and perampanel have been tested on the amoebas (Apparasamy et al., 2025). Additionally, both formulations substantially mitigated host cell cytopathogenicity, offering dual benefits of amoebicidal and protective effects. However, the observed dose-dependent cytotoxicity toward mammalian cells highlights the need for careful therapeutic calibration. Future investigations should prioritize *in vivo* validation, surface engineering for targeted delivery, and pharmacokinetic profiling to enhance BBB penetration. Altogether, this work advances the concept of nanotechnology-enhanced drug re-purposing as a viable strategy for addressing the urgent challenges in treating neuro-invasive amoebic infections (Meunier et al., 2025)

## 5 Conclusion

This paper demonstrated the promising therapeutic potential of repurposed CNS drugs conjugated with AgNPs against *A.castellanii* and *N. fowleri* which would respectively afflict GAE and PAM. The nanoconjugates exhibited potent amoebicidal activity, inhibited key survival mechanisms such as encystation and excystation, and significantly reduced host cell cytopathogenicity. While both formulations showed moderate cytotoxicity toward human cell lines, their ability to penetrate the BBB and exert species-specific effects highlights their value as dual-action agents. These findings support further *in vivo* studies and optimization strategies to enhance safety and efficacy, paving the way for nanotechnology-driven solutions to combat neuroinvasive amoebic infections.

## 6 Future studies

Although this study establishes the *in vitro* efficacy of Zonisamide-AgNPs and Perampanel-AgNPs against *A. castellanii* and *N. fowleri*, future research should prioritize *in vivo* validation using appropriate animal models to evaluate pharmacokinetics, permeability of the BBB and therapeutic results under physiological conditions. These studies will be critical in determining the safety, biodistribution, and long-term effects of the nanoconjugates.

Additionally, transcriptomic profiling through mRNA sequencing (RNA-seq) should be employed to elucidate the molecular mechanisms underlying amoebicidal activity. Differential gene expression analysis can identify key genes that are upregulated or downregulated in response to treatment, offering insights into stress response pathways, apoptosis, encystation inhibition, and potential resistance mechanisms. For instance, upregulation of oxidative stress markers or downregulation of encystation-related genes could validate the proposed modes of action. Such data would not only strengthen mechanistic understanding but also guide the rational design of next-generation nanotherapeutics with enhanced specificity and reduced host toxicity.

## CRediT authorship contribution statement

**Devandran Apparasamy:** Writing – review & editing, Writing – original draft, Visualization, Validation, Project administration, Funding acquisition, Data curation, Conceptualization. **Niwasini Krishna Kumar:** Writing – review & editing, Methodology, Data curation. **Chris Izaak Jones:** Writing – review & editing, Software, Methodology, Investigation, Data curation. **Yee Mon Htet:** Writing – review & editing, Software, Investigation, Data curation. **Haema Thevanayagam:** Writing – review & editing, Resources, Investigation, Formal analysis. **Usman Ahmed:** Writing – review & editing, Methodology, Investigation. **Mohd Farooq Shaik:** Writing – review & editing, Resources, Investigation, Formal analysis. **Ayaz Anwar:** Writing – review & editing, Resources, Formal analysis. **Kavitha Rajendran:** Writing – review & editing, Validation, Supervision, Resources, Project administration, Conceptualization.

## Ethics approval and consent to participate

This study did not involve human participants or live animals. All experiments were performed on established human cell lines (HaCaT and SH-SY5Y) obtained from recognized repositories and cultured *in vitro*. The use of these cell lines and pathogenic amoebae (*Acanthamoeba castellanii* ATCC 50492 and *Naegleria fowleri* ATCC 30174) complied with the institutional biosafety and ethical standards of Sunway University. No additional ethical approval was required.

## Consent for publication

Not Applicable

## Funding

This work was supported by Sunway University, Malaysia under the internal grant scheme (GRTIN-RAG-DADTP-03-2024).

## Declaration of competing interest

The authors declare that they have no known competing financial interests or personal relationships that could have appeared to influence the work reported in this paper.

## Data Availability

All data generated or analyzed during this study are included in this published article. Additional datasets are available from the corresponding author on reasonable request.
